# Bi-Force: large-scale bicluster editing and its application to gene expression data biclustering

**DOI:** 10.1093/nar/gku201

**Published:** 2014-03-20

**Authors:** Peng Sun, Nora K. Speicher, Richard Röttger, Jiong Guo, Jan Baumbach

**Affiliations:** 1 Max Planck Institute for Informatics, Campus E1 4, Saarland University, 66123 Saarbrücken, Germany; 2 Cluster of Excellence for Multimodel Computing and Interaction, Campus E1 7, Saarland University, 66123 Saarbrücken, Germany; 3 Institute for Mathematics and Computer Science, University of Southern Denmark, Campusvej 55, 5230 Odense M, Denmark

## Abstract

The explosion of the biological data has dramatically reformed today's biological research. The need to integrate and analyze high-dimensional biological data on a large scale is driving the development of novel bioinformatics approaches. Biclustering, also known as ‘simultaneous clustering’ or ‘co-clustering’, has been successfully utilized to discover local patterns in gene expression data and similar biomedical data types. Here, we contribute a new heuristic: ‘Bi-Force’. It is based on the weighted bicluster editing model, to perform biclustering on arbitrary sets of biological entities, given any kind of pairwise similarities. We first evaluated the power of Bi-Force to solve dedicated bicluster editing problems by comparing Bi-Force with two existing algorithms in the BiCluE software package. We then followed a biclustering evaluation protocol in a recent review paper from Eren *et al.* (2013) (A comparative analysis of biclustering algorithms for gene expressiondata. *Brief. Bioinform.*, 14:279–292.) and compared Bi-Force against eight existing tools: FABIA, QUBIC, Cheng and Church, Plaid, BiMax, Spectral, xMOTIFs and ISA. To this end, a suite of synthetic datasets as well as nine large gene expression datasets from Gene Expression Omnibus were analyzed. All resulting biclusters were subsequently investigated by Gene Ontology enrichment analysis to evaluate their biological relevance. The distinct theoretical foundation of Bi-Force (bicluster editing) is more powerful than strict biclustering. We thus outperformed existing tools with Bi-Force at least when following the evaluation protocols from Eren *et al.* Bi-Force is implemented in Java and integrated into the open source software package of BiCluE. The software as well as all used datasets are publicly available at http://biclue.mpi-inf.mpg.de.

## INTRODUCTION

The enormous amount of available biological data from laboratories around the world has greatly re-shaped today's biological studies. GenBank alone stores over 197 000 000 sequences of more than 380 000 organisms (1); the Gene Expression Omnibus (GEO), a public database of gene expression data, provides approximately 1 million samples, 40 000 series, and 3500 datasets for more than 11 000 microarray platforms (2). Integrating, processing and analyzing large quantities of data from various such ultra large data sources has become one of the big bioinformatics challenges.

Clustering is commonly accepted as a powerful approach to explore gene expression datasets (3). Given a pairwise similarity function transformed into a similarity matrix, clustering algorithms seek to partition the data items into a list of disjoint groups, such that the similarities within each group are maximized and those between different groups are minimized. Traditional clustering approaches cluster only rows or columns in one run, which is not always beneficial (4). In contrast, biclustering allows to ‘simultaneously’ partition both rows and columns. If we are given, for instance, gene expression datasets for different cellular conditions, biclustering is more powerful in capturing biologically meaningful subsets of condition-specific genes. The major reason is that the expression of gene subsets may correlate only under some conditions while being independent under other conditions. Biclustering approaches are generally capable of discovering such local patterns. They have proven particularly useful for various types of gene expression data analysis (5) but should also work on other omics datasets, proteomics or metabolomics, for instance (6).

The first such ‘biclustering’ tool was developed by Cheng and Church and applied to gene expression data (7). Since then, many other biclustering tools have been reported (e.g. 8–12) and been suggested for applications to various biomedical problems (13,14). Biclustering tools became increasingly popular due to their ability to simultaneously cluster biological data from different sources in order to discover local bi-correlation patterns. Several systematic comparisons have been published using various measurements to evaluate a number of prevailing biclustering tools on both synthetic and real-world datasets (15–17).

### Bicluster editing

A direct connection between biclustering and graph theory can be established by converting the biclustering problem to a node partition problem on bipartite graphs: ‘weighted bicluster editing’. To specify the problem, first a data matrix needs to be transformed into a ‘weighted bipartite graph’, denoted as *G* = (*U*, *V*, *E*), where *U* and *V* are the two sets of nodes and *E* is the edge set with each edge having exactly one end vertex in *U* and the other end vertex in *V*.

Given a matrix *M* describing the similarity between the elements from *U* and *V* (gene expression of different genes under different conditions, for instance). For each row *r*_*i*_, a node *u*_*i*_ ∈ *U* is created; for each column *c*_*j*_, a node $v$_*j*_ ∈ *V* is created. The pairwise similarity between two arbitrary nodes *u*_*i*_ ∈ *U* and $v$_*j*_ ∈ *V* is computed by a system- or user-defined similarity function based on the corresponding element *a*_*ij*_ in the matrix, i.e. *s*(*u*_*i*_$v$_*j*_) = *f*(*a*_*ij*_). A user-specified threshold ‘*t*_0_’ is then used to judge if *u*_*i*_ and $v$_*j*_ are connected (if *s*(*u*_*i*_$v$_*j*_) ≥ *t*_0_) or not (if *s*(*u*_*i*_$v$_*j*_) < *t*_0_). An undirected simple bipartite graph *G* = (*U*, *V*, *E*) is called a ‘biclique’ if for all *u* ∈ *U* and $v$ ∈ *V*, we have *uv* ∈ *E* (*s*(*uv*) ≥ *t*_0_). A bicluster graph is a graph where every disjoint component is a biclique. In the weighted bicluster editing model, which is later utilized for biclustering, our goal is to edit the given input graph *G* = (*U*, *V*, *E*) by deleting and inserting edges in such a way that it becomes a bicluster graph. Each deletion or insertion incurs a non-negative cost: if *uv* ∈ *E*, then the edge deletion cost is cost(*uv*) = *s*(*uv*) − *t*_0_. If *uv* ∉ *E*, then the edge insertion cost is cost(*uv*) = *t*_0_ − *s*(*uv*). The cost for a set of edge insertions or deletions *S* is thus defined as: cost(*S*) = ∑_*uv* ∈ *S*_cost(*uv*). Therefore, the weighted bicluster editing model can be described as: given a weighted bipartite graph *G* = (*U*, *V*, *E*) and a similarity function *s*(*uv*), can *G* be transformed into a bicluster graph *G*′ = (*U*, *V*, *E*′) by edge insertions and deletions with the total cost cost(*G* → *G*′) = cost(*E*\*E*′) + cost(*E*′\*E*) minimized?

The counterpart of bicluster editing on general graphs is ‘cluster editing’. It is one of the classic NP-complete problems, and it has been extensively studied, both theoretically (18–20) and in practical applications (21,22). A recent overview may be found in (23). Bicluster editing, though less studied, has been proven NP-complete as well (24). A number of exact and approximate algorithms have been developed to solve the problem (13, 25). Given the sizes of nowadays real-world datasets and given the theoretical problem complexity, however, there is no hope to efficiently solve this problem exactly.

Here, we present a software implementing a novel heuristic algorithm that efficiently solves the weighted bicluster editing problem: Bi-Force. It comes as an extension of BiCluE software package. We previously developed BiCluE to provide ‘exact’ solutions for the (weighted) bicluster editing problem, sufficient for small-scale and medium-scale problem instances (13). The Bi-Force extension that we describe in the following part of this paper is dedicated to solve the large-scale problem instances that we face in nowadays bioinformatics more and more frequently. First, we compare the novel Bi-Force with the two existing exact fixed-parameter algorithm (FPA) and the edge-deletion heuristics (EDH). After showing that Bi-Force solves the suggested bicluster editing model sufficiently well on real data, we will demonstrate the model's appropriateness for biomedical biclustering problems. Therefore, we will follow the recently suggested evaluation protocols from Eren *et al.*, who analyzed in their paper several existing biclustering tools regarding their performance on gene expression data (17). Bi-Force will be compared to eight existing biclustering software implementations on (i) artificial datasets generated with six different models and (ii) Gene Ontology enrichment analysis of nine real gene expression datasets for mouse, worm and human, extracted from the GEO database, just as in the review paper from (17).

## MATERIALS AND METHODS

We first describe the data that we used for evaluation. Afterward, we outline the algorithmic approach behind Bi-Force. Finally, we introduce the existing software and the evaluation routine.

### Artificial data

#### Artificial graphs

Artificial graphs were generated for two purposes: (i) parameter training and (ii) evaluation of Bi-Force to other BiCluE algorithms. Each artificial graph with *n* vertices was created by randomly assigning the pairwise similarities based on the following rules: randomly pick up *k* (*k* ∈ [1, *n*]) nodes and define them to be in one bicluster. This step was repeated on the remaining *n* − *k* nodes until no node was left. Similarities were computed with two Gaussian distributions: $N(\mu
                _{{\rm intra}},\sigma ^2_{{\rm intra}})$ and $N(\mu _{{\rm inter}},\sigma
                ^2_{{\rm inter}})$. The first one was used to assign the similarities between two nodes belonging to the same pre-defined bicluster (intra-bicluster similarities), and the latter was used to assign the similarities between two nodes from different pre-defined biclusters (inter-bicluster similarities). We adjusted the parameters in the Gaussian distributions to control the ‘error-edge-rate’, i.e. the probability of the occurrence of ‘intra missing edges’ (missing edges within a pre-defined bicluster) or an ‘inter-edge’ (edge between two different pre-defined biclusters). 0 was chosen to be the edge threshold *t*_0_. A set of such bipartite graphs with varying error-edge-rates was created: from ‘almost-bicluster’ (error-edge-rate is equal to 0.14) graphs to fully random graphs (error-edge-rate is equal to 0.5). ‘Almost-bicluster’ graphs with relative low error-edge-rates were used to simulate real-world biological networks, which usually need only a small number of edge modifications to make a bipartite graph into a bicluster graph. An increased error-edge-rate means that the input graph is more ‘mixed-up’ and thus requires more edge insertions and deletions. To evaluate the bicluster editing algorithms (Bi-Force and two existing BiCluE algorithms), we assessed their robustness for input graphs varying from ‘almost-bicluster’ to ‘mixed-up’ error-edge-rates.

#### Synthetic data matrices

For a comprehensive comparison of the performance between Bi-Force and eight other biclustering tools, we created synthetic data matrices based on six different models. Each synthetic data matrix consists of 300 rows and 200 columns, within which a pre-defined bicluster with 30 rows and 30 columns was randomly selected. For each of the following models, 10 data matrices were generated for simulation repetition. With this strategy we generally followed the protocol suggested by Eren *et al.* (17).

- Constant biclusters: the values in the matrix of randomly selected 30 rows × 30 columns bicluster were set to a constant expression level of 0. The background values, i.e. the elements in the matrix that are not within the pre-defined bicluster were chosen randomly but independently from Gaussian distribution *N*(0, 1).
- Constant-upregulated biclusters: as in the previous model but the expression levels in the 30 × 30 bicluster were fixed to 5, i.e. simulating constant upregulation.
- Shift-scale biclusters: before generating each data matrix, a base row *R*_*b*_ = {*a*_*b*, 1_, *a*_*b*, 2_, …, *a*_*b*, 200_} was chosen. For every row *r*_*i*_ in the pre-defined bicluster, a scale factor α_*i*_ and a shift factor β_*i*_ were randomly generated. Each element *a*_*ij*_ in the pre-defined bicluster was both shifted and scaled from the base row: *a*_*ij*_ = α_*i*_ · *a*_*bj*_ + β_*i*_. The selected rows in the pre-defined bicluster could be positively or negatively shifted (or scaled), depending on the values of the shift (or scale) factors. The elements in base row and background were drawn independently from Gaussian distribution *N*(0, 1). All scale factors and shift factors were drawn independently from distribution *N*(3, 1).
- Shift biclusters: similar to the shift-scale model, but with fixed scale factors of α_*i*_ = 1.
- Scale biclusters: similar to shift-scale model, but with fixed shift factors of β_*i*_ = 0.
- Plaid biclusters: this model is an additive bicluster model, first introduced in (26). Each matrix element is modeled as the sum of several different effects, including background effect θ, cluster effect μ, row effect α, and column effect β:
  \begin{equation*} a_{ij} = \theta +\sum _{k=1}^{K} (\mu
                    _k+\alpha _{ik}+\beta _{jk})\rho _{ik}\kappa _{jk}, \end{equation*}
  where *a*_*ij*_ is the element in the matrix, and ρ and κ are the indicators for the membership in bicluster *k* for row *i* and column *j*, respectively. All effects were independently and identically distributed according to the Gaussian distribution *N*(0, 1).

### The Bi-Force algorithm

The main methodological contribution of this paper is an algorithm that heuristically solves the weighted bicluster editing problem. Bi-Force is motivated by the well-known physics-inspired graph layout algorithm of Fruchterman and Reingold (27). It mainly seeks to arrange all nodes of a graph in a two-dimensional plane such that ‘similar’ nodes are located more close to each other than to others. Bi-Force, afterward, assigned the nodes from each ‘dense’ part of the graph layout to one bicluster by single-linkage clustering (SLC) or *k*-means clustering based on the Euclidean distances. The algorithm is carried out in a three-step procedure: (i) layout generation, (ii) bicluster partitioning, and (iii) post-processing.

#### Layout generation

In this stage, the coordinates of all nodes are generated and re-arranged in a way that the nodes with higher similarities are located next to each other, and far away from those that are dissimilar. Bi-Force computes pairwisely the ‘physical forces’ between two nodes, i.e. the magnitudes that similar nodes attract each other, dragging them closer while dissimilar nodes repulse each other, pushing them farther away. The whole algorithm starts with an initial layout where nodes are ‘almost’ evenly located on a two-dimensional circle with randomly permuted order. The radius *R* of the circle is a parameter of Bi-Force. The strength of attracting/repulsing force depends on the current positions of the two nodes, attraction/repulsion coefficient and the corresponding cost to delete the edge or to insert the missing edge between the two nodes. The re-arrangement is performed in an iterative manner. In each round, the movement of each node is the cumulative effect of the attractions and repulsions from all other nodes. Afterward, all nodes are re-positioned to the new locations simultaneously according to the magnitudes of the movements. The whole procedure is repeated for *I* times. The attracting/repulsing effect from node $v$ to *u* is computed by the following formula:

\begin{equation*}
                f_{u\leftarrow v} = \left\lbrace \begin{aligned} \frac{{\rm cost}(uv)\cdot f_{{\rm
                att}} \cdot {\rm log}(d(u,v)+1) }{|V|} \ \ \ \text{for attraction}\\ \frac{{\rm
                cost}(uv) \cdot f_{{\rm rep}}}{|V| \cdot {\rm log}(d(u,v) +1)} \ \ \ \ \ \ \text{for
                repulsion}. \\ \end{aligned} \right. \end{equation*}

In the formula above, *f*_*u*_ ← $v$ represents the attracting/repulsing effect from node $v$ to *u*, i.e. the magnitude of the movement of *u* caused by $v$. When there is an edge between *u* and $v$, they attract each other and if otherwise, they repulse. *f*_*att*_ and *f*_*rep*_ are the attractive and repulsive factors, respectively. *d*(*u*, $v$) represents the Euclidean distance between node *u* and $v$. Obviously, the threshold *t*_0_ affects the density/granularity of the bicluster editing model: the smaller *t*_0_ is, the fewer biclusters there are and the larger their sizes, and vice versa.

To accelerate the convergence of the nodes to stable positions, a cooling parameter is used to limit the maximal magnitudes of attractions and repulsions. This means in a certain iteration *i*, the movement magnitude cannot exceed the current cooling parameter *M*_*i*_. Cooling parameter starts with an initial value *M*_0_ as a parameter in Bi-Force and decreases with every iteration.

At the end of this stage, the positions of all nodes are fixed and similar nodes should be close to each other. In the next step, we make use of this assumption and partition the layouted graph in a way that optimizes the editing costs.

#### Bicluster partitioning

Based on the coordinates of the nodes obtained in the previous stage, we partition the graph into disjoint biclusters using two different geometric clustering methods: SLC and *k*-means. Both SLC and *k*-means are standard methods in computational cluster analysis (28). The density parameters of the two algorithms (distance threshold δ for SLC and the number of clusters *k* for *k*-means) are varied systematically (SLC: δ = 0…*M*_0_ + *R* in steps of σ, *k*-means: *k* = 2…|*V*|/3). For each clustering result, we compute the necessary editing costs to create this solution. Finally, we keep the solution that has minimal editing costs before we proceed to post-processing.

#### Post-processing

Here, we try to further reduce the clustering costs, which includes two steps: (i) bicluster merging and (ii) nodes moving.

To reduce the number of redundant biclusters, particularly the singletons, we try to merge biclusters. First, all the biclusters are ordered by size in an ascending order. Let *B* = (*b*_1_, *b*_2_, …, *b*_*l*_) be the *l* ordered biclusters, where |*b*_*i*_| ≤ |*b*_*j*_|, for all *i* ≤ *j*. For all pairs of biclusters *b*_*i*_ and *b*_*j*_ with 1 ≤ *i* < *j* ≤ *l*, we calculate the cost that would emerge from merging the two, i.e. cost(*b*_1_, *b*_2_, …, *b*_*i*_∪*b*_*j*_, …, *b*_*l*_). Once a *B*′ with a lower overall cost than before is found, we re-define the biclusters according to *B*′ by merging *b*_*i*_ and *b*_*j*_. This step is repeated until no beneficial merging can be done anymore.

After merging the clusters, another post-processing step similar to Restricted Neighborhood Search Clustering (29) is carried out. Let ${B=(b_{1},b_{2},...,b_{l}{\prime}})$ be the biclusters after the merging step, for each *b*_*i*_ and *b*_*j*_, such that 1 ≤ *i* < *j* ≤ *l*, we compute the costs that would emerge from moving $v$ ∈ *b*_*i*_ to *b*_*j*_. If the overall cost can thereby be reduced in this step, $v$ is moved to *b*_*j*_. Similarly, this step is repeated until no vertex move is beneficial.

This is the final result of the Bi-Force algorithm. The details of Bi-Force algorithm can be found in the Supplementary File 3. The software implementation's output is a list of nodes together with their bicluster memberships and their final layout positions. For each instance, we also report the number of editing actions (edge insertion and deletions) as well as the total cost to compute this solution.

#### Analysis

The worst-case running time of Bi-Force is dependent on the three steps mentioned above. Let *n* = |*U*| + |*V*| for an input graph *G* = (*U*, *V*, *E*). In the ‘layout generation’ step, where Bi-Force arranges the positions of all nodes, it consumes *O*(*n*^2^) time to compute the mutual attracting/repulsing forces in each iteration. Thus, the layout generation step finishes in *O*(*I* · *n*^2^), where *I* is the number of iterations. The SLC runs in *O*(*D*_1_ · *n*^2^), where *D*_1_ is the number of different thresholds used. The *k*-means problem is by its nature NP-hard (30,31). However, we limited the maximal number of iterations in *k*-means to be 200 and thus it finishes in *O*(200 · *n*) time. Finally, for post-processing, each iteration takes *O*(*n*^2^) time and the total running time is bounded by *O*(*D*_2_ · *n*^2^) for *D*_2_ iterations. Since *D*_2_ might increase with *n*, we added an empirical limit of 500 iterations to *D*_2_. In most cases, Bi-Force did not reach this limit and we observed only small numbers of iterations before it terminated.

In summary, the overall running time for Bi-Force grows quadratic in the number of nodes.

#### Training

Bi-Force is a heuristic algorithm that has several parameters in the algorithm requiring to be optimized: the number of iterations *I*, the attraction and repulsion coefficients, *f*_*att*_ and *f*_*rep*_, the initial maximum magnitude *M*_0_ and the radius for initial layout *R*. Hence, two evolutionary training strategies were implemented: a general training procedure and an input-specific parameter training.

In ‘general training’, we tried to find a set of parameters that fits a ‘general scenario’, i.e. graphs with varying error-edge-rates. Graphs for general training were generated according to the protocol described in the ‘Artificial Data’ section. By varying the deviations of the two Gaussian distributions, graphs with nine different error-edge-rates were generated: 0.10, 0.15, 0.20, 0.25, 0.30, 0.35, 0.40, 0.45, and 0.50, with 10 repeats for each error-edge-rate resulting in a training graph set of 90 graphs.

The training was conducted in an evolutionary manner: first, we randomly selected 1000 parameter sets within certain ranges: (0, 10) for *f*_*att*_ and *f*_*rep*_, (0, 300) for the iterations *I*, (0, 1000) for initial maximum magnitude *M*_0_, and (0, 400) for radius *R*. Then we applied the randomly selected 1000 parameter sets on our artificial graph set and picked the best three parameter sets (minimal total costs). These sets were used as a starting point for the following training procedure.

One training iteration consists of two steps: (i) for each parameter set, compute the sum of the costs for solving all graphs; (ii) generate new parameter sets in an evolutionary manner based on the old sets and their costs. After running the algorithm on all input graphs, the parameter sets were ordered ascendingly by total costs. New list of parameters for the next training iteration was generated based on the first three parameter sets with least costs in the previous iteration: we kept the two best parameter sets and put them directly in the new parameter list. The third parameter set in the new list was computed as the mean of the best three parameter sets in the previous iteration. Then, the best three sets were permuted to obtain the fourth, fifth and sixth sets in the new list. The next three sets for the new iteration were randomly picked up around the best set in the previous iteration. For each parameter α (α: *f*_att_, *f*_rep_, *M*_0_, *R* and *I*), we randomly picked up a number within the range of (0.9α, 1.1α). Finally, in a similar way, we randomly picked the last three sets in the new list, only altering the ranges to (0, 2α). The emerging 12 new parameter sets entered the next iteration of training. Then the whole procedure was repeated. After a given number of iterations, we picked up the best set of parameters as the final optimized set.

Besides general training, for each input case, an additional ‘specific training’ (ST) was conducted to further refine our best general parameter set to fit to each specific graph inputs. We make use of the following trick: without loss of generality, a bicluster editing problem instance is assumed to contain only one connected component, since disjoint components can be treated separately without interfering the results of other components. Real biological data often contain more than one such connected component. We further assume that smaller components have a similar graph ‘structure’ as the larger ones. Once an input case is given, we train the input-specific parameters on the smaller disjoint components in order to ‘adapt’ our algorithm to the specific input data without great compromise of the running time (as smaller instances can be computed faster than bigger ones). The whole procedure works as follows: all the connected components of a given input graph are sorted in accordance to their sizes. Then, the parameter set, optimized from the general training, is further trained on the small disjoint components in the input. We start with the smallest components, following the same evolutionary training procedure as in the general training. On the second-smallest component, we repeat this process but with less training iterations (*T*_*max*_ − 0.5 × size of the component). We stop this parameter training when a connected component size of *T*_*max*_ is reached (here we use *T*_*max*_ = 40) and apply the best parameter set found so far to all bigger problem instances.

The initial parameter combination obtained from general training is: *I* = 134, *f*_*att*_ = 2.484, *f*_*rep*_ = 1.323, *M*_0_ = 51.84 and *R* = 112.5. We used this combination as the starting point for ST, as described above.

#### Bi-Force for biclustering

As mentioned above, data matrices of gene expression datasets may be seen as weighted bipartite graphs. Thus, biclustering problems may be solved by solving the bicluster editing model. In order to extract biclusters with different features (e.g. over-expressed bicluster, under-expressed biclusters, etc.), Bi-Force provides four biclustering scenarios: (a) Over-expressed scenario, (b) under-expressed scenario, (c) low-deviated scenario and (d) high-deviated scenario. Given a matrix *M*, in all four scenarios, a bipartite graph *G* = (*U*, *V*, *E*) is constructed as described before: For each row *r*_*i*_, a node *u*_*i*_ ∈ *U* is created and for each column *c*_*i*_, a node $v$_*i*_ ∈ *V* is created. We achieve different biclustering scenarios by varying the similarity functions. In the over-expressed scenario where higher expression values are to be extracted, a user-defined threshold *t*_0_ must be given and all expression levels above *t*_0_ are considered as ‘over-expressed’. The similarity between two arbitrary nodes *u*_*i*_ and $v$_*j*_ is set directly to be the corresponding element in matrix, *s*(*u*_*i*_$v$_*j*_) = *a*_*ij*_. Similarly, in the under-expressed scenario, *t*_0_ is given to seek for the ‘under-expressed’ expression values. The similarity function is then set to be *s*(*u*_*i*_$v$_*j*_) = 2*t*_0_ − *a*_*ij*_ to cluster the lower-expressed genes and conditions. For rest two scenarios, two user-specified parameters must be given: ‘data center’ *t*_*c*_ and ‘deviation threshold’ *t*_0_. In low-deviated scenario, the similarity function is defined as: *s*(*u*_*i*_$v$_*j*_) = 1/(|*a*_*ij*_ − *t*_*c*_| + 1), where the less *a*_*ij*_ is deviated from *t*_*c*_, the larger *s*(*u*_*i*_$v$_*j*_) is, to cluster low-deviated regions in the matrix. In high-deviated scenario, similarities are defined as: *s*(*u*_*i*_$v$_*j*_) = |*a*_*ij*_ − *t*_*c*_| to search for farther deviated elements. In all four scenarios, an edge is drawn between *u*_*i*_ and $v$_*j*_ if and only if the corresponding *s*(*u*_*i*_$v$_*j*_) ≥ *t*_0_. In addition, Bi-Force allows the user to filter noisy bicluster results in two ways, by using a rank parameter *k*_*r*_ or a size parameter *k*_*s*_. If *k*_*r*_ is given, Bi-Force only outputs the largest *k*_*r*_ biclusters in the result set. For *k*_*s*_, Bi-Force outputs all biclusters with sizes larger than *k*_*s*_ − 1. All biclusters not satisfying the criteria are removed from the result.

### Comparison against two bicluster editing algorithms

The performance of Bi-Force on bicluster editing problems is assessed by comparing it with two other algorithms in the package BiCluE: FPA and EDH. Two experiments were conducted to compare the accuracy of the three algorithms in terms of editing costs and to assess the robustness of Bi-Force. For accuracy evaluation, 80 artificial graphs with various sizes but constant small error-edge-rate (arbitrarily chosen as 0.14) were generated. For robustness assessment, however, we fixed the sizes of input graphs to be 80 and varied the error-edge-rate (0.1, 0.15, 0.2, 0.25, 0.3, 0.35 and 0.4). EDH and Bi-Force were applied on the inputs to test their capacity of keeping low running time while error-edge-rates increase. The running times for each graph input were limited to 2 h.

### Comparison against eight biclustering algorithms

To evaluate the performance of Bi-Force on biclustering problems, we referenced to the work of Eren *et al.* (17). Eight (out of twelve) prevalent online available biclustering algorithms were downloaded, including Cheng and Church (7), BiMax (16), FABIA (32), ISA (33), Plaid (26), QUBIC (34), Spectral (35) and xMOTIFs (36). Five of the eight methods are integrated in the R package ‘biclust’. The three remaining software packages (FABIA, ISA and QUBIC) were downloaded from the project web sites. Four other tools were not included in this study since no corresponding online resources are available or errors exist in the programs. Note that the omitted algorithms are not among the best-performing tools in the study of Eren *et al.* The details of the biclustering algorithms including the references and the important parameters influencing the performances of the algorithms are listed in Table 1.

**Table 1.** The applied biclustering tools and their parameter space

Algorithm	References	Parameters
Bi-Force	–	Edge threshold: *t*_0_.
FABIA	(32)	Number of biclusters: *p*.
QUBIC	(34)	Range of possible ranks: *r*;
Percentage of regulating conditions for each gene: *q*;		
Number of biclusters: *p*.		
Cheng and Church	(7)	Variance threshold: δ;
Multi-deletion parameter: α.		
Plaid	(26)	Number of max. iterations for each layer: *M*_*I*_;
Max. number of layers: *M*_*L*_.		
BiMax	(16)	Number of biclusters: *n*;
Min. row size: *minr*;		
Min. column size: *minc*.		
Spectral	(35)	Normalization method: *norm*;
Min. row size: *minr*;		
Min. column size: *minc*.		
xMOTIFs	(36)	Number of biclusters: *n*.
ISA	(33)	Number of seeds: *n*_*s*_.

### Parameters

Appropriate parameter setting is crucial to the performance of each algorithm. Algorithms cannot simply be applied with default parameters as not all of them fit all bicluster analysis scenarios. We carefully optimized the parameters of each tool such that they show their best performances on both, the synthetic data as well as the gene expression data.

For the synthetic datasets, all algorithms that require a user-given number of biclusters were given the correct number. For gene expression data, the number was set to be 50 biclusters. Parameters other than ‘number of biclusters’ were optimized through performance on synthetic datasets, i.e. we tried various parameters (or combinations of parameters) for each algorithm and took the parameter (or combination of parameters) that could achieve the best performance. Particularly, for the algorithms requiring more than one parameter, a grid-search strategy was implemented: a number of candidate values were chosen empirically for each parameter and we compared the performances of the algorithm with every possible combination of the parameters on synthetic datasets. We give details about the utilized parameter space of each tool in Supplementary File 1.

### Evaluation on synthetic data

The performance of all biclustering algorithms on synthetic data was evaluated by comparing the set of result biclusters against the pre-defined biclusters. As suggested in the work of Eren *et al.* (17), we chose the Jaccard coefficient to compute the similarity of two different biclusters. Let *b*_1_ and *b*_2_ be two biclusters, we define:

\begin{equation*} s(b_1,b_2)
              = \frac{|b_1 \cap b_2|}{|b_1 \cup b_2|} , \end{equation*}

where |*b*_1_∪*b*_2_| and |*b*_1_∩*b*_2_| are the number of nodes in the union and intersection of *b*_1_ and *b*_2_, respectively. Obviously, the largest value of Jaccard coefficient is 1 when *b*_1_ and *b*_2_ are identical and the lowest value 0 is reached when two sets are disjoint. It can be interpreted as the percentage of shared elements of two biclusters.

For two sets of biclusters, the pre-defined set of biclusters *T* (true set) and the result set of biclusters *R* (from the nine algorithms), we calculated two scores: recovery and relevance scores, defined to quantify the similarities between *T* and *R*. Recovery score indicates the percentage of the truth set that is found in the result. It is maximized when *T*⊆*R*. Similarly, relevance score represents the percentage of the result set that is overlapped with the true biclusters. It is maximized when *R*⊆*T*. Formally,

\begin{equation*} {\rm Recovery}: S(T,R) = \frac{1}{|T|} \sum
              _{b_1\in T} \max _{b_2\in R} s(b_1,b_2) \end{equation*}

\begin{equation*} {\rm Relevance}:S(R,T) = \frac{1}{|R|} \sum _{b_1\in R} \max
              _{b_2\in T} s(b_1,b_2). \end{equation*}

Again, note that we are in coherence with Eren *et al.* here.

### Evaluation on real gene expression data

For gene expression data, a different evaluating method must be used since true biclusters are unknown *a priori*. We validated the results by computing Gene Ontology (GO) term enrichments for all the biclusters. Principle Component Analysis imputation was used to compute the missing values in the gene expression datasets (37). Enrichment analysis was carried out by using GOstats (38) on three categories (biological process ontology, molecular function ontology and cellular compartment ontology). In hypergeometric tests, genes within each bicluster were used as the input vector, and genes involved in the gene expression study were used as the gene universe. Afterward, multiple test correction was performed to adjust the *p*-values by using the method from Benjamini and Hochberg (39). A bicluster was considered ‘enriched’ in a certain GO category if any adjusted *p*-value of any GO term was smaller than *p* = 0.05. Again, we agreed and followed Eren *et al.*'s suggestions with this protocol.

## RESULTS AND DISCUSSION

### Comparison against two bicluster editing algorithms

In this section, the performance of Bi-Force and two bicluster editing algorithms (FPA and EDH) was compared in three aspects: editing costs (accuracy), running times and robustness. We used two different datasets: almost-bicluster graphs and graphs with various error-edge-rates. Almost-bicluster graphs were generated with an error-edge-rate of 0.14 but various sizes ranging from 20 to 140 nodes. The three algorithms were applied on all graphs, while recording the editing costs and the running times. Maximum running time was set to 2 h. Table 2 shows the results. Although Bi-Force assigns random initial positions of all nodes, the final outputs are surprisingly stable over different runs. The stability of Bi-Force was tested on all artificial graphs and always the same results were given. Due to combinatorial explosion, FPA was only able to finish the inputs for graphs smaller than 50 nodes.

**Table 2.** Running times and editing costs of the bicluster editing algorithms

		Bi-Force No S.T.		Bi-Force S.T.		EDH		FPA	
Vertices	Edges	Costs	R.T.	Costs	R.T.	Cost	R.T.	Costs	R.T.
20	[20–36]	95.17	**0.21**	92.70	55.15	109.40	0.076	**86.94**	2.10
25	[30–49]	173.61	0.236	169.90	129.12	228.77	**0.17**	**165.27**	84.35
30	[46–89]	252.49	**0.31**	247.61	131.70	350.43	0.405	**241.27**	233.61
35	[47–115]	363.52	**0.40**	365.95	329.66	378.155	0.77	**347.18**	949.34
40	[86–114]	540.74	**0.52**	517.85	272.19	667.69	1.19	**510.86**	912.648
50	[142–204]	908.24	**0.79**	891.87	366.38	961.19	8.37	**880.74**	1523.21
60	[273–335]	1510.30	**1.10**	1510.30	1.17	1549.06	49.58	**1498.30**	3160.32
70	[223–438]	1853.43	**1.56**	1853.43	1.66	**1852.086**	73.32		
80	[313–509]	**2348.18**	**1.98**	**2348.18**	2.064	2449.92	307.21		
90	[417–641]	3252.69	2.54	3252.69	2.56				
100	[525–1220]	3833.84	3.29	3833.84	3.11				
110	[526–1378]	4840.47	3.91	4840.47	3.86				
120	[770–1573]	5621.08	4.62	5621.08	4.60				
130	[807–1773]	6928.51	5.71	6928.51	5.76				
140	[890–1440]	7327.50	6.84	7327.50	6.85				

The cells belonging to the same algorithm in the first row are merged as suggested by the reviewer. Vertical lines added performance comparison between Bi-Force and two existing bicluster editing algorithms. The results here are the average of five repeated runs. EDH stands for edge-deletion heuristics, FPA stands for fixed-parameter algorithm, S.T. stands for specific training (of Bi-Force's heuristic parameters), and R.T. is the run time (given in s). The smallest editing cost and running time are marked with bold font. Note that when the sizes of the input graphs grew larger than 100 nodes, no specific training was conducted such that the two Bi-Force variants gave the same results. Execution of all tools was interrupted after 2 h running time without termination.

**Table 3.** GDS datasets

Dataset	Genes	Samples	Description
GDS181	12 559	84	Gene expression profiles from diverse tissues, organs and cell lines with normal physiological state.
GDS589	8799	122	Examination of normal physiological gene expression in 11 peripheral and 15 brain regions in three common out-bred rat strains.
GDS1027	15 866	154	Sulfur mustard effect on lungs: dose response and time course.
GDS1319	22 548	123	Various C blastomere mutant embryos analyzed to deconvolve C blastomere lineage-specific expression patterns specified by the PAL-1 homeodomain protein.
GDS1406	12 422	87	Analysis of seven brain regions of six inbred strains of mouse.
GDS1490	12 422	150	Mouse neural tissue profiling.
GDS2225	15 923	6	Mechanical strain effect on fetal lung type II epithelial cells.
GDS3715	12 559	110	Insulin effect on human skeletal muscle.
GDS3716	22 215	42	Breast cancer: histologically normal breast epithelium.

The EDH with its polynomial running times is much faster than the FPA. As shown in Table 2, Bi-Force without ST of the parameters is fastest. With ST enabled running time increases slightly. Note that ST was performed only for smaller problem instances (up to 50 nodes; see algorithm description above). Thus, running times and costs are the same afterward, since ST was switched off. Bi-Force is generally fastest, followed by EDH and the exact FPA. As FPA is an exact algorithm, it always came with the smallest editing costs. For larger problem instances, FPA did not terminate anymore within 2 h. Here Bi-Force performed better than EDH in most cases.

**Figure 1.**
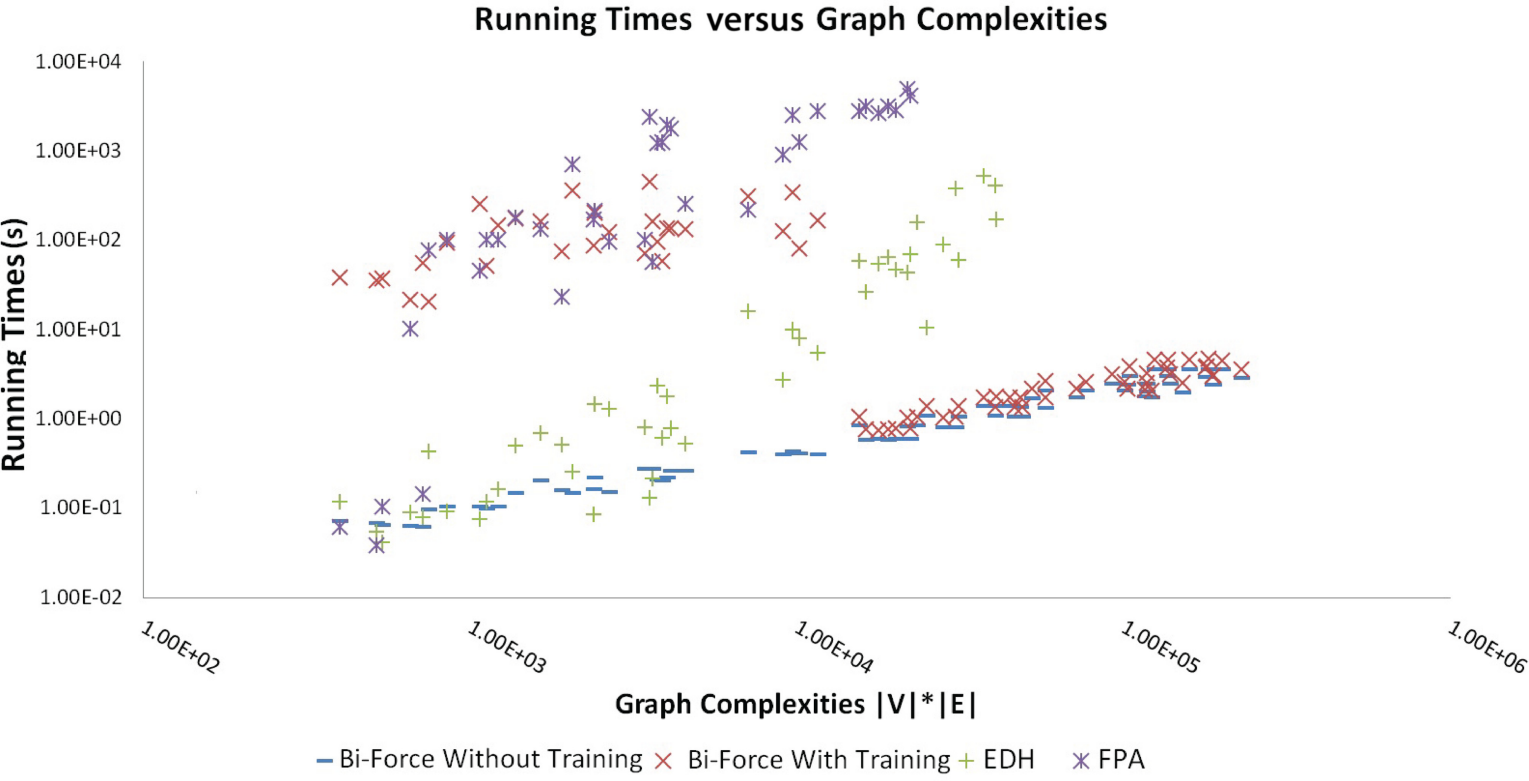
Running times against graph complexities. The running times are plotted against the graph complexities of the input instances (|*V*| · |*E*|). Note the effect of the specific training (ST) of parameters, which is turned off for larger graphs (see text).

We also compared the running times against graph complexities (see Figure 1). Here ‘graph complexity’ refers to the product of the number of nodes and the number of edges in a given graph. Clearly, Bi-Force outperforms the two existing algorithms.

**Figure 2.**
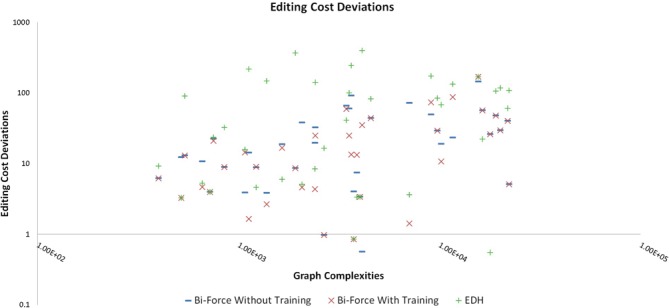
Deviations (in editing costs) from the optimal solution of the FPA algorithm.

The accuracy of Bi-Force (with and without ST) is plotted against that of the EDH heuristic as function of the cost deviations, i.e. the differences in editing costs between the heuristic (EDH and Bi-Force) and the exact algorithms (FPA) in Figure 2 for smaller graphs (where FPA terminated). Bi-Force clearly achieved better overall editing costs than EDH. With standard parameter set obtained from general training, Bi-Force managed to achieve smaller costs than EDH. Nevertheless, in many cases, Bi-Force with ST returned solutions with less costs. This justifies our strategy to evolutionarily train the heuristic parameters on small problem instances (where this can be achieved fast) and relying on the assumption that a parameter set will work for larger connected components of the same graph similarly well.

**Figure 3.**
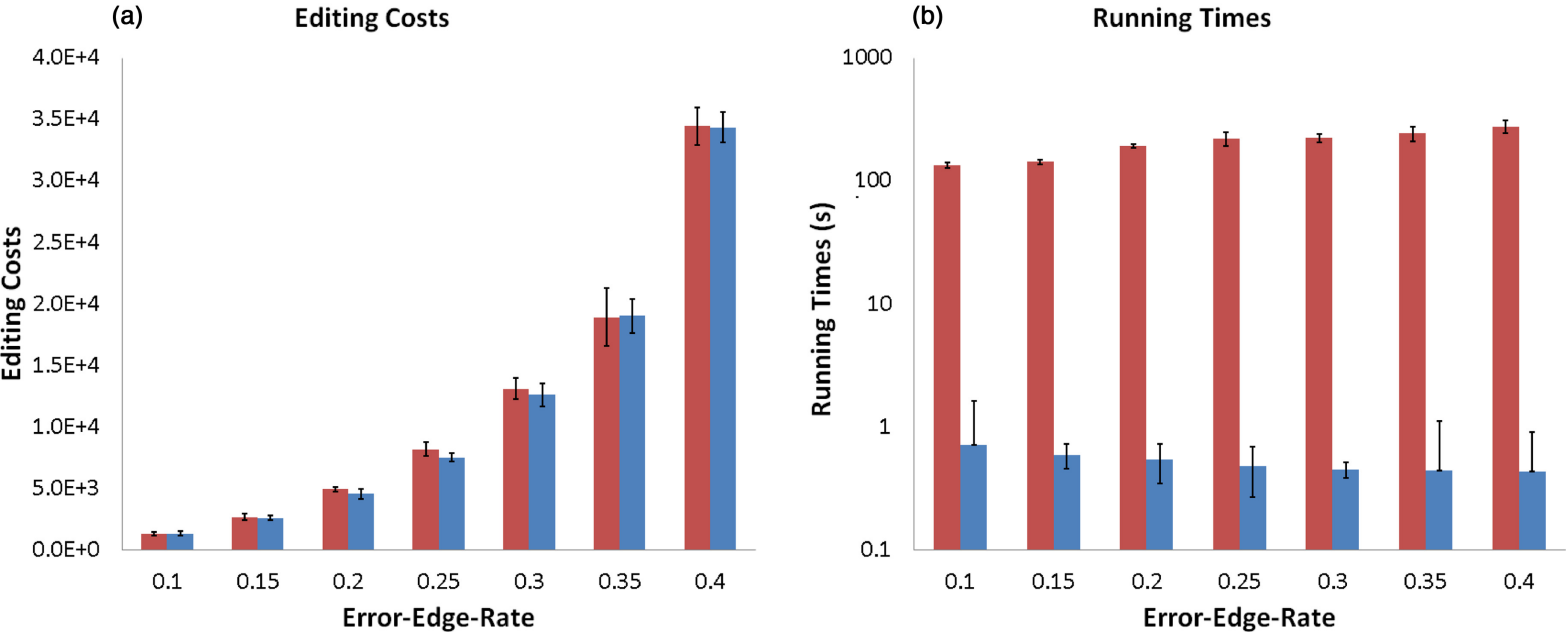
Error bars added. Robustness of the edge-deletion heuristics and Bi-Force. Input graphs were generated with different error-edge-rates and the running times were measured. Note the *log*-scales of the y-axes.

Figure 3 illustrates the robustness of Bi-Force to varying error-edge-rates. Artificial graphs with seven different error-edge-rates were generated, with 10 repeats for each rate. We now compare the editing costs and the running times of Bi-Force and EDH on these artificial datasets. Figure 3a shows, as expected, that with increase of error-edge-rates, the editing costs for both algorithms increase as well, polynomially. Figure 3b shows that the run times of both tools are generally quite robust toward changing graph structures and the running time correlates only with the input sizes.

### Comparison against eight biclustering algorithms

#### Artificial data

**Figure 4.**
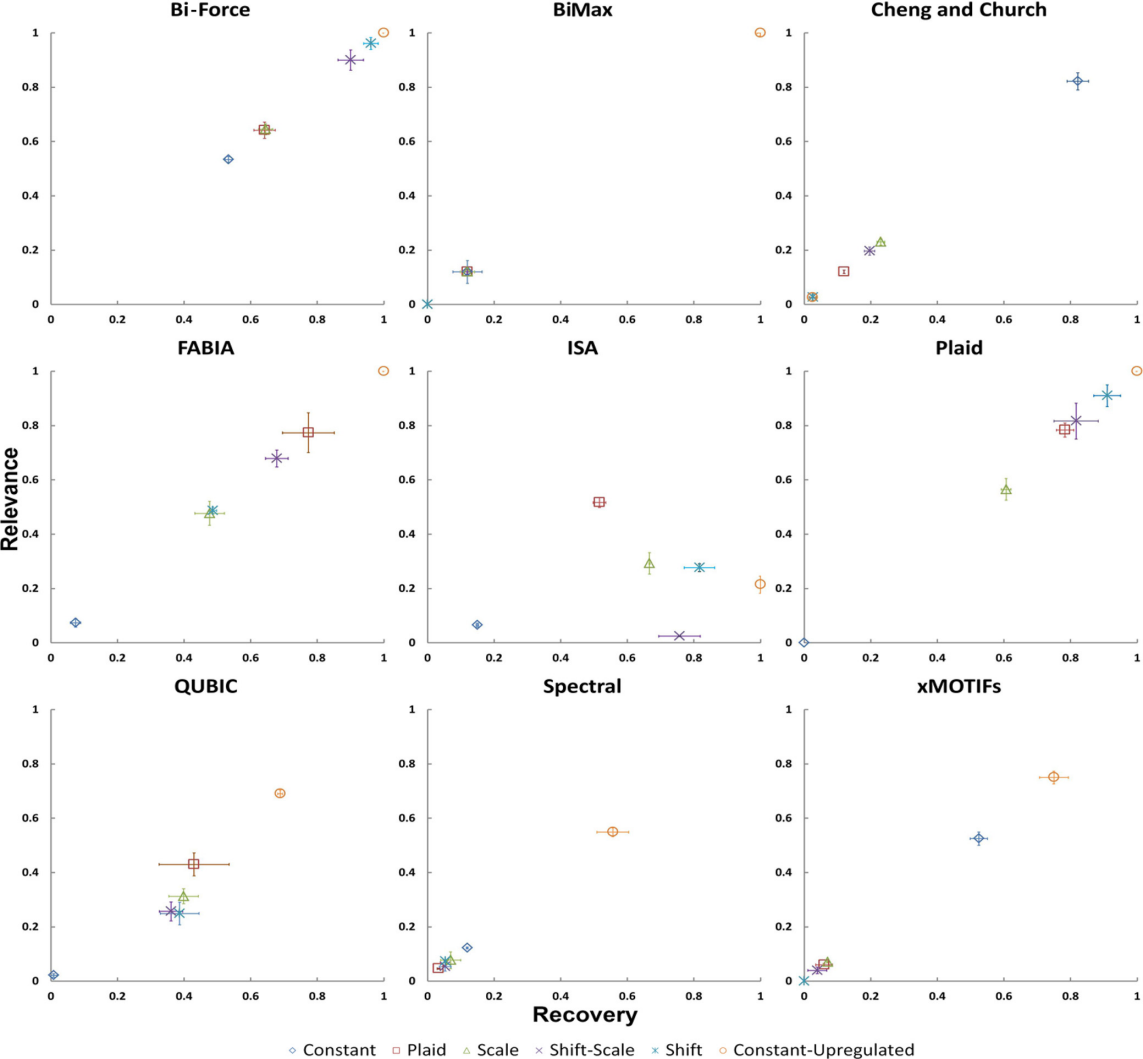
Error bars added. The comparison of Bi-Force against eight existing biclustering algorithms on synthetic datasets. Each plot includes the average recovery versus relevance of datasets from five different data sampling models (see text).

**Figure 5.**
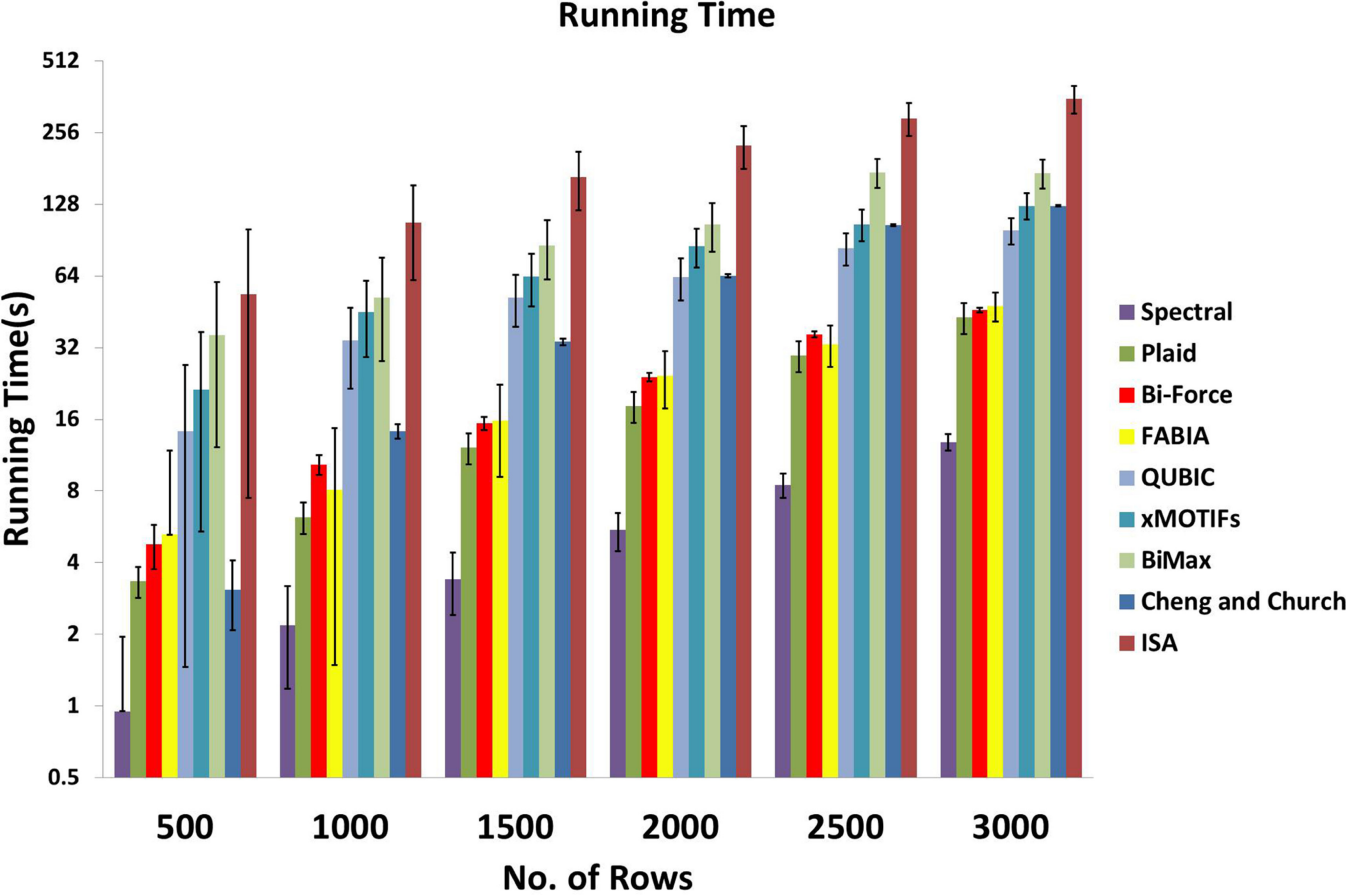
Error bars added. Running times of the nine algorithms for increasing number of rows in the expression matrix. The y-axis is in log-scale.

Given that Bi-Force, as shown above, solves the ‘bicluster editing model’ well enough, we now seek to apply this model to biclustering of biological datasets. As mentioned many times before, we follow the evaluation protocol published in a recent review paper from Eren *et al.* (17). In Figure 4, we compare the ‘relevance’ and ‘recovery’ of Bi-Force as well as the above introduced existing tools. Figure 5 compares the running times of all the algorithms with inputs of fixed columns of 300 and rows of varying sizes.

We believe the bicluster editing model underlying Bi-Force to be more robust regarding different dataset types compared to the existing biclustering algorithms. The main assumption behind bicluster editing, and thus Bi-Force, is that the average similarities within the biclusters are above the user-given threshold while the mean similarities between elements from different biclusters is below the threshold. This way, the threshold as single density parameter controls the size and granularity of the biclustering results. If Bi-Force is configured to output only the largest bicluster, it seeks the largest sub-matrix in the dataset with significant difference between elements inside the bicluster compared to the background. Thus, Bi-Force successfully recovered all the biclusters for the constant-upregulated model. For the inputs of shift and shift-scale model, since elements inside the bicluster were shifted by a certain magnitude, Bi-Force was also able to recover most of (∼85–95%) the pre-defined biclusters. In the scale model where data elements were comparatively weakly shifted from the background, the results were a little bit worse but still over half of the pre-defined biclusters were recovered (∼60–70% for the scale model). For Plaid model where most of the elements were generated only based on the ‘background effect’, we conducted biclustering to extract ‘high-deviated’ data and over 60% of the pre-defined biclusters were discovered. For the constant model, we tried to cluster the data elements with ‘low-deviated’ values and ∼55–60% of the pre-defined biclusters were successfully captured. A brief discussion of the other tools’ performance may be found in Supplementary File 2.

#### Gene expression data

We now continue with the protocol from Eren *et al.* and apply all nine algorithms to real-world biological data: gene expression microarray data from the GEO database (GDS181, GDS589, GDS1027, GDS1319, GDS1406, GDS1490, GDS2225, GDS3715 and GDS3716; see Table 3 for a summary). Their performance was evaluated by means of GO term enrichment analysis.

**Figure 6.**
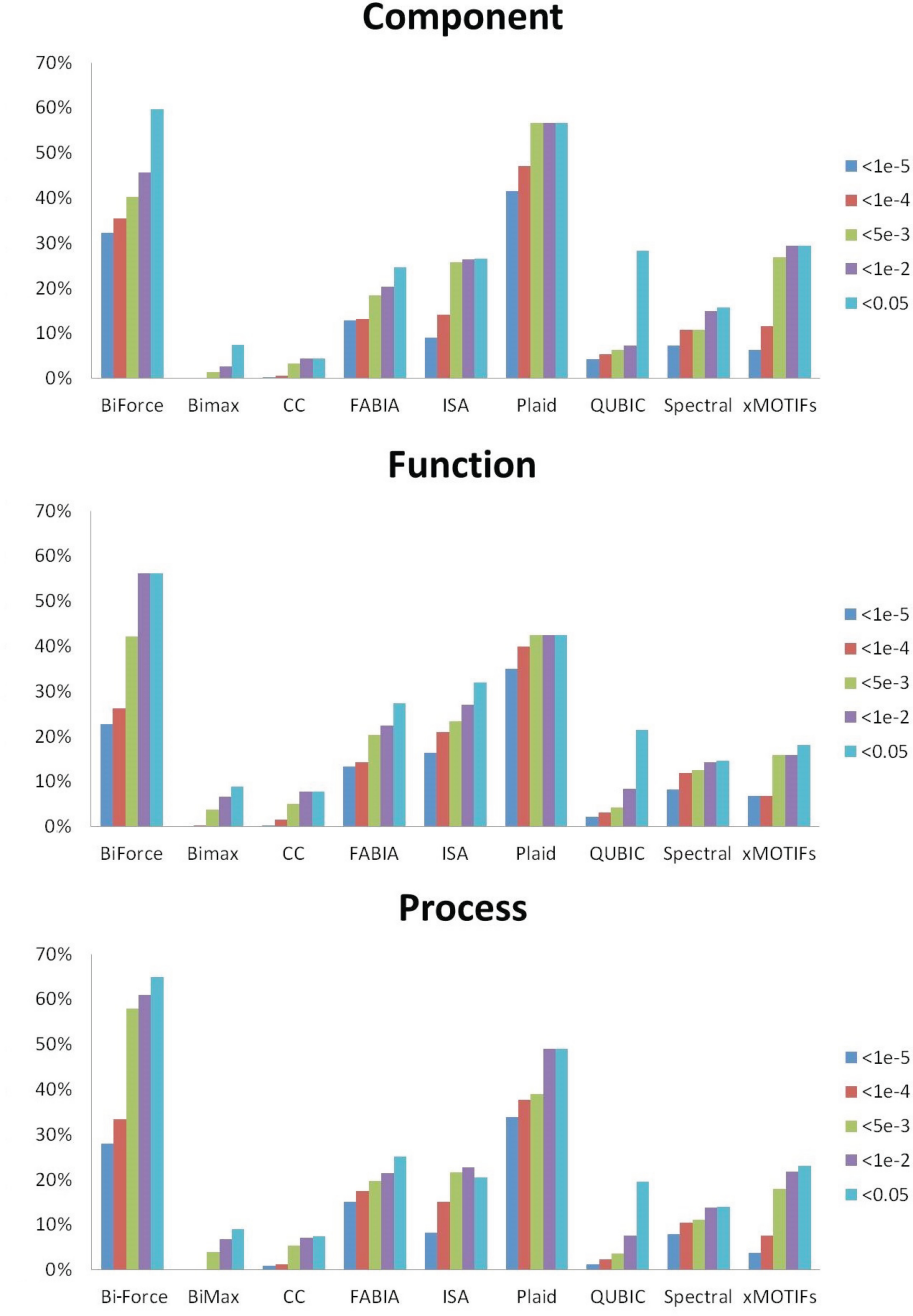
Proportions of GO-enriched biclusters for different algorithms on five significance level (see text).

Before GO term enrichment analysis was performed, the biclusters identified by the nine algorithms were further filtered to decrease redundancy: biclusters with more than 80% overlap were removed. Afterward, GO term enrichment analysis was conducted on the filtered biclusters for all three categories (biological process, molecular function and cellular compartment). Table 4 shows the number of enriched biclusters in all three categories. Figure 6 gives the proportions for different significance levels of the biclusters found by all algorithms. BiMax found the most biclusters, however most of them were not enriched at reasonably high *p*-value cut-offs. Thus the average enrichment level for BiMax is comparably low. Similarly, Cheng and Church, QUBIC and Spectral have similar problems with high numbers of false positives. In contrast, most of the biclusters found by Bi-Force and Plaid are highly enriched. Although xMOTIFs also provided many enriched biclusters, it did not find any biclusters for the datasets GDS1027, GDS1319 and GDS3715. Bi-Force clearly outperformed the other tools as in average approximately 55% of the reported biclusters are also enriched with high *p*-value confidence cut-offs, more than with the competing eight tools. For completeness, the four GO terms with lowest *p*-values for each category are given in Supplementary Table 1.

**Table 4.** The results of GO enrichment analysis, including the numbers of reported biclusters and the numbers of enriched biclusters

Algorithm	Found	Enriched (%)
Bi-Force	129	76(58.91%)
FABIA	189	47(24.87%)
QUBIC	873	200(22.91%)
Cheng and Church	1962	107(5.45%)
Plaid	180	87(48.33%)
BiMax	2439	205(8.41%)
Spectral	1095	161(14.70%)
xMOTIFs	339	79(23.30%)
ISA	261	67(25.67%)

The proportions of enriched biclusters reported by Bi-Force support our conclusion that the bicluster editing model is a well-working formulation for biclustering. However, the numbers of biclusters discovered by Bi-Force is comparably low. This might be because Bi-Force is no fuzzy partitioning approach such that by definition all identified biclusters are independent of each other. In future implementations, we will enable Bi-Force to search for overlapping biclusters by adding a strategy that utilizes two density thresholds.

## CONCLUSION

We have presented Bi-Force, the yet fastest software for solving the bicluster editing problem. We demonstrated its flexibility by applying it to biclustering, a restricted version of bicluster editing with many applications in gene expression data analysis. We compared it to eight existing tools by following an established evaluation protocol from Eren *et. al.*'s review paper. We show that Bi-Force outperformed the existing tools on synthetic datasets and on real-world gene expression data. Last but not the least, we wish to emphasize that Bi-Force has the ability to perform simultaneous clustering of arbitrary multiple datasets. It is not restricted to gene expression scenarios. Instead, any types of biological data that can be modeled as bipartite graph can be partitioned by using Bi-Force. It is now part of the BiCluE software package and publicly available at http://biclue.mpi-inf.mpg.de.

## Supplementary Material

Supplementary Data

SUPPLEMENTARY DATA
