# Supplementary material for: Bi-Force: large-scale bicluster editing and its application to gene expression data biclustering
Source: Nucleic Acids Res. 2014 Mar 20;42(9):e78. doi: 10.1093/nar/gku201 (PMC5769343; doi:10.1093/nar/gku201)
Supplement: Supplementary Data [file gku201_supplementary_data.zip › nar-02990-met-n-2013-File012.pdf]

SUPPLEMENTARY FILE 1.

Bi-Force only requires the threshold to model the edges in the bipartite graphs generated from matrices. For each synthetic data, we tried 10 thresholds, from $\frac{19}{20}e$ to $\frac{1}{2}e$, where e is the difference between the maximum and the minimum values in the matrix, decreasing $\frac{1}{20}e$ each time. Then the thresholds with the best performance were used. For expression data sets, where no gold-standard result is present,t_0 was set to be $\frac{9}{10}e$,

For the Cheng and Church algorithm, where δ controls the maximum variances in the biclusters and α regulates the speed of the algorithm. We implemented grid-search strategy with δ ranging from 0.1 to 2.5 and α from 1.5 to 3. Based on the performance, we chose $\delta = 0.1$ and $\alpha = 1.5$ for synthetic data sets. However, for the gene expression data sets which are much larger, $\delta = 0.1$ seems to be over-stringent, then we took an empirically-beneficial value of $\delta = e/2000$ (**?**). α was decreased from 1.5 to 1.1 to avoid over-slimming the biclusters.

For Bimax and Spectral which require minimum row and column sizes, a grid-search was conducted in the ranges from 2 to 20 for both rows and columns and finally 10 was chosen to be the minimum sizes for rows and columns in a bicluster. Moreover, for Spectral algorithm, we compared the performances of different normalization methods and finally chose "logarithmic normalization" for both synthetic and gene expression data sets.

Two important parameters largely influenced the performance of QUBIC: the range of the possible ranks r and the percentage of regulating conditions for each gene q. As suggested by the author, we conducted the grid-search, starting from a relatively small value of r, from 1 to the half of the number of columns in the matrix, which was 100. For q, we set our range from 0.02 to 0.08, centered by the default value 0.06. Afterwards we found the default values (1 for r and 0.06 for q) worked the best on synthetic data sets. The values of both parameters were kept the same for gene expression data sets.

Note that two algorithms (Bimax and xMOTIFS) require discretized data, thus the input matrices were all binarized into 0s and 1s, using the means of the corresponding matrices as thresholds.

For ISA algorithms, we tested different numbers of seeds, from 100 to 400 and chose 200 seeds for synthetic data. For gene expression data sets, which are expected to be more complex, we increased the number of seed to 400.

SUPPLEMENTARY FILE 2.

FABIA performed best on the constant-upregulated data sets, followed by the performance on shift-scale and plaid data sets. For the other data sets, less than half of the real biclusters were found because FABIA is optimized to perform better if the distribution of the data set is highly unsymmetric (**?**). If the values in the data sets are symmetrically distributed or have a Gaussian-like distribution, then the performances of FABIA suffer. (**?**)

QUBIC recovered most of the constant-upregulated biclusters. It also successfully recovered part of the biclusters of scale data sets, shift-scale data sets and plaid data sets.

The Cheng and Church algorithm was expected to find biclusters with low mean square residues. It performed well on the constant data set. With over 80% of the pre-defined biclusters recovered it is the best among the nine algorithms on the constant model. However, for all the other models with data shifted from the background, the qualities of the results of Cheng and Church decrease significantly.

Plaid successfully identified most of the biclusters within constant-upregulated, shift, shift-scale, scale and plaid model. It achieved recovery and relevance scores almost as good as Bi-Force. However, no bicluster was found for the constant model, indicating a poor performance of Plaid to extract constant biclusters.

BiMax bi-discretizes the data elements in the matrix by using a given threshold. This over-simplifies many scenarios. Thus BiMax performed well only on constant-upregulated data where biclusters were largely shifted away from the background. For all the other models, BiMax's performances were relatively poor.

Similarly, xMOTIFs discretizes the data and thus only biclusters for the constant model were well recovered.

Spectral clustering, though the fastest tool, has a comparatively weak overall performance. Even for the constant-upregualted data sets, only about 60% of the true biclusters were recovered.

ISA recovered most of the biclusters in all the models but constant model. However, ISA generated a number of redundant biclusters that lowered overall relevance scores. A post-running filter merging the highly overlapping biclusters might be beneficial for ISA.

SUPPLEMENTARY FILE 3.

Algorithm - Bi-Force

Input: Similarity matrix A ($a_{ij} \in A$, $1 \leq i \leq m$, $1 \leq j \leq n$), a threshold t.
Parameter: Number of iterations I, attractive factor f_{att}, repulsive factor f_{rep}, cooling parameter M_0 and the radius of the initial layout R.
Result: The biclusters discovered by Bi-Force.

▷ Two vectors store the coordinates of the nodes in two sets
$pos_1 = (pos_1[1], ..., pos_1[m])$ ▷ The coordinates for the nodes in set 1.
$pos_2 = (pos_2[1], ..., pos_2[n])$ ▷ The coordinates for the nodes in set 2.

initNodePos(pos_1,pos_2) ▷ Initialize the positions of all nodes.

for $iter = 1$ TO I **do**

$disp_1[] = array[m]$
$disp_2[] = array[n]$ ▷ Initialize the two movement vectors for all nodes.

for $i = 1$ TO m **do**
for $j = 1$ TO n **do** ▷ Compute the movement vectors
if $a_{ij} \geq t$ **then**
$f_{i \leftarrow j} = \log(d(i,j)+1) \cdot (a_{ij} - t) \cdot f_{att}/(m+n)$
▷ Compute attraction force. $d(i,j)$ is the Euclidean distance.
else
$f_{i \leftarrow j} = 1/\log(d(i,j)+1) \cdot (a_{ij} - t) \cdot f_{rep}/(m+n)$
▷ Compute compulsion force. $d(i,j)$ is the Euclidean distance.
end if
$disp_1[i] += f_{i \leftarrow j} \cdot (pos_1[i] - pos_2[j])/d(i,j)$
$disp_2[j] -= f_{i \leftarrow j} \cdot (pos_1[i] - pos_2[j])/d(i,j)$
end for
end for
for $i = 1$ TO m **do** ▷ Move the nodes
$disp_1[i] = disp_1[i] \cdot \min\{(M(iter)/|disp_1[i]|), 1\}$
$pos_1[i] += disp_1[i]$
end for
for $j = 1$ TO n **do** ▷ Move the nodes
$disp_2[j] = disp_2[j] \cdot \min\{(M(iter)/|disp_2[j]|), 1\}$
$pos_2[j] += disp_2[j]$
end for
end for
$biclusters = \operatorname{argmin}\{cost(\text{SLC}(pos_1, pos_2)), cost(\text{kmeans }(pos_1, pos_2))\}$ ▷ Run Single-linkage clustering and Kmeans
$biclusters =$postprocessing$(biclusters)$
return $biclusters$
