# Supplementary material for: Bi-Force: large-scale bicluster editing and its application to gene expression data biclustering
Source: Nucleic Acids Res. 2014 Mar 20;42(9):e78. doi: 10.1093/nar/gku201 (PMC5769343; doi:10.1093/nar/gku201)
Supplement: SUPPLEMENTARY DATA [file supp_42_9_e78__index.html]

Bi-Force: large-scale bicluster editing and its application to gene expression data biclustering — SUPPLEMENTARY DATA 

# Bi-Force: large-scale bicluster editing and its application to gene expression data biclustering

## SUPPLEMENTARY DATA

**Files in this Data Supplement:**

- SUPPLEMENTARY DATA
- SUPPLEMENTARY DATA
